# 2,6-Bis(1*H*-benzimidazol-2-yl)pyridine butyric acid monosolvate dihydrate

**DOI:** 10.1107/S1600536812021915

**Published:** 2012-05-19

**Authors:** Songzhu Lin, Ruokun Jia, Aimin He

**Affiliations:** a Northeast Dianli University, Jilin 132012, People’s Republic of China

## Abstract

In the title compound, C_19_H_13_N_5_·C_4_H_8_O_2_·2H_2_O, the mol­ecular skeleton of the 2,6-bis­(benzimidazol-2-yl)pyridine (bbip) mol­ecule is essentially planar (r.m.s. deviation = 0.023 Å). An extensive three-dimensional network of inter­molecular N—H⋯O, O—H⋯O and O—H⋯N hydrogen bonds consolidates the crystal packing, which also exhibits π–π inter­actions between the five- and six-membered rings from neighbouring bbip mol­ecules.

## Related literature

For background to supra­molecular inter­actions, see: Dale *et al.* (2004 ▶); Braga *et al.* (2005 ▶); Ring *et al.* (2006 ▶). For related structures, see: Freire *et al.* (2003 ▶); Xiao *et al.* (2010 ▶).
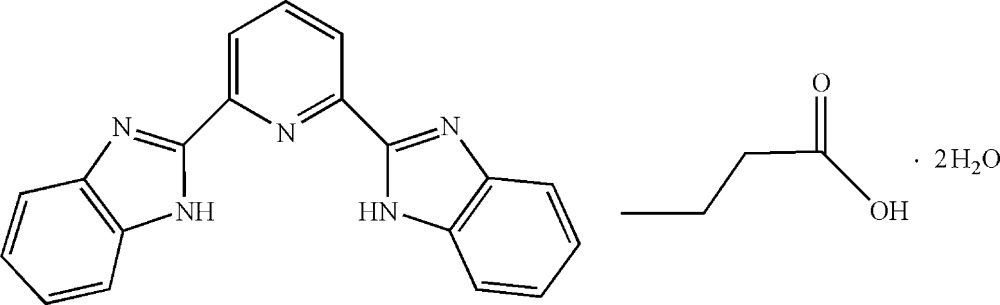


## Experimental

### 

#### Crystal data

C_19_H_13_N_5_·C_4_H_8_O_2_·2H_2_O
*M* *_r_* = 435.48
Triclinic, 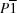

*a* = 9.3950 (19) Å
*b* = 9.5611 (19) Å
*c* = 13.805 (3) Å
α = 103.27 (2)°
β = 99.91 (3)°
γ = 104.76 (3)°
*V* = 1131.5 (4) Å^3^
*Z* = 2
Mo *K*α radiation
μ = 0.09 mm^−1^
*T* = 295 K
0.20 × 0.18 × 0.15 mm

#### Data collection

Enraf–Nonius CAD-4 diffractometer
Absorption correction: ψ scan (North *et al.*, 1968 ▶) *T*_min_ = 0.982, *T*_max_ = 0.987
8683 measured reflections
3981 independent reflections
3195 reflections with *I* > 2σ(*I*)
*R*_int_ = 0.019
3 standard reflections every 100 reflections intensity decay: none

#### Refinement

*R*[*F* ^2^ > 2σ(*F* ^2^)] = 0.045
*wR*(*F* ^2^) = 0.144
*S* = 1.10
3981 reflections
318 parameters
6 restraints
H atoms treated by a mixture of independent and constrained refinement
Δρ_max_ = 0.22 e Å^−3^
Δρ_min_ = −0.25 e Å^−3^

### 

Data collection: *CAD-4 Software* (Enraf–Nonius, 1989 ▶); cell refinement: *CAD-4 Software*; data reduction: *NRCVAX* (Gabe *et al.*, 1989 ▶); program(s) used to solve structure: *SHELXS97* (Sheldrick, 2008 ▶); program(s) used to refine structure: *SHELXL97* (Sheldrick, 2008 ▶); molecular graphics: *SHELXTL* (Sheldrick, 2008 ▶); software used to prepare material for publication: *WinGX* (Farrugia, 1999 ▶).

## Supplementary Material

Crystal structure: contains datablock(s) global, I. DOI: 10.1107/S1600536812021915/cv5296sup1.cif

Structure factors: contains datablock(s) I. DOI: 10.1107/S1600536812021915/cv5296Isup2.hkl

Supplementary material file. DOI: 10.1107/S1600536812021915/cv5296Isup3.cml

Additional supplementary materials:  crystallographic information; 3D view; checkCIF report

## Figures and Tables

**Table 1:** Hydrogen-bond geometry (Å, °)

*D*—H⋯*A*	*D*—H	H⋯*A*	*D*⋯*A*	*D*—H⋯*A*
N1—H2⋯O2*W*	0.90 (2)	2.12 (1)	3.006 (2)	166 (2)
N5—H1⋯O2*W*	0.86 (2)	2.234 (19)	3.083 (2)	169.1 (17)
O1*W*—H2*W*1⋯N4	0.83 (2)	1.96 (1)	2.7901 (19)	178 (3)
O2*W*—H2*W*2⋯O1*W*^i^	0.82 (2)	2.04 (1)	2.852 (2)	168 (3)
O2*W*—H1*W*2⋯O1*W*^ii^	0.81 (2)	2.06 (1)	2.856 (2)	168 (3)
O1*W*—H1*W*1⋯O1^iii^	0.82 (2)	2.00 (1)	2.795 (2)	166 (3)
O2—H3⋯N2	0.85 (2)	1.89 (1)	2.712 (2)	164 (2)

Symmetry codes: (i) 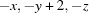; (ii) 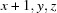; (iii) 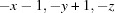.

**Fig. 1.**
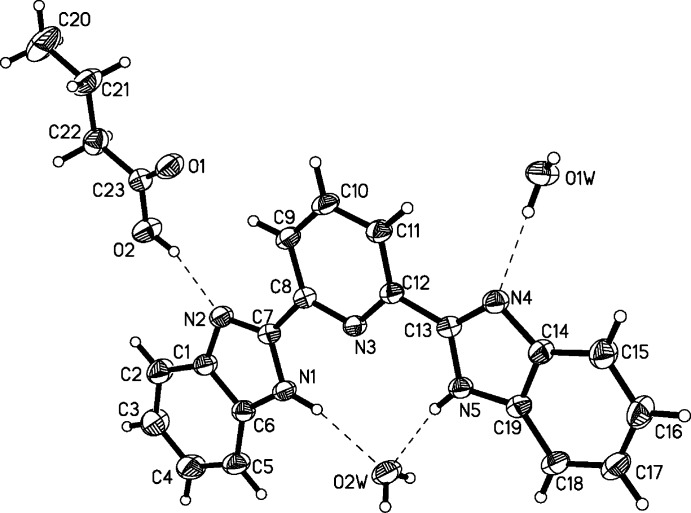
View of (I) showing 30% probability displacement ellipsoids and the atom-numbering scheme. Dashed lines indicate hydrogen bonds.

## supplementary crystallographic information

### Comment

Crystal engineering of extended frameworks can readily be achieved using a variety of supramolecular interactions (Braga *et al.*, 2005; Ring *et al.*, 2006). However, the prediction of the solid state packing of simple organic cocrystals still remains an ongoing challenge (Dale *et al.*, 2004). The supramolecular interactions, such as hydrogen bonding and π-π stacking interactions, can create network materials with infinite 1-D, 2-D or 3-D structural motifs. The supramolecular structures with 2,6-bis(benzimidazol-2-yl)pyridine have been reported in recent year (Freire *et al.*, 2003; Xiao *et al.*, 2010). As a continuation of those works, we report the crystal structure of the title compound (I).

In (I) (Fig. 1), all bond lengths and angles are normal and correspond to those observed in the related structures (Freire *et al.*, 2003; Xiao *et al.*, 2010). The aromatic C—C and C—N bond lengths in both the benzimidazole and pyridine rings are within the usual range. All C and N atoms in the 2,6-bis(benzimidazol-2-yl)pyridine molecule are almost coplanr with the largest deviation of 0.060 Å for C4.

In the crystal, the N—H···O, N—H···O and O—H···O hydrogen bonds (Table 1) consolidate the packing, which exhibits π–π interactions with the following center-to-center distances - *Cg*1···*Cg*1=3.625 (2) Å and *Cg*2···*Cg*3=3.775 (2) Å, where *Cg*1, *Cg*2 and *Cg*3 are centroids of N4/C13/N5/C19/C14, C14—C19 and N3/C8—C12, respectively.

### Experimental

2,6-Bis(benzimidazol-2-yl)pyridine (0.062 g, 0.20 mmol) and butyric acid (0.018 g, 0.20 mmol) were dissolved in 30 ml solution mixed with ethanol and water by 2:1(V/V), then heated to refluxed for 6 h and cooled to the room temperature. Single crystals suitable for X-ray measurements were obtained by recrystallization at room temperature.

### Refinement

N- and O-bound H atoms were found in a difference Fourier map, and isotropically refined with the restraints (O—H = 0.82 (2), 0.85 (2) Å; N—H = 0.88 (2) Å). C-bound H atoms were fixed geometrically (C—H = 0.93–0.97 Å), and refined as riding, with *U*_iso_(H) =1.2–1.5 *U*_eq_(C).

### Crystal data

**Table:**

C_19_H_13_N_5_·C_4_H_8_O_2_·2H_2_O	*Z* = 2
*M**_r_* = 435.48	*F*(000) = 460
Triclinic, *P*1	*D*_x_ = 1.278 Mg m^−^^3^
Hall symbol: -P 1	Mo *K*α radiation, λ = 0.71073 Å
*a* = 9.3950 (19) Å	Cell parameters from 25 reflections
*b* = 9.5611 (19) Å	θ = 4–14°
*c* = 13.805 (3) Å	µ = 0.09 mm^−^^1^
α = 103.27 (2)°	*T* = 295 K
β = 99.91 (3)°	Block, colourless
γ = 104.76 (3)°	0.20 × 0.18 × 0.15 mm
*V* = 1131.5 (4) Å^3^	

### Data collection

**Table:**

Enraf–Nonius CAD-4 diffractometer	3195 reflections with *I* > 2σ(*I*)
Radiation source: fine-focus sealed tube	*R*_int_ = 0.019
Graphite monochromator	θ_max_ = 25.0°, θ_min_ = 3.1°
ω scans	*h* = −11→11
Absorption correction: ψ scan (North *et al.*, 1968)	*k* = −11→10
*T*_min_ = 0.982, *T*_max_ = 0.987	*l* = −16→16
8683 measured reflections	3 standard reflections every 100 reflections
3981 independent reflections	intensity decay: none

### Refinement

**Table:**

Refinement on *F*^2^	Secondary atom site location: difference Fourier map
Least-squares matrix: full	Hydrogen site location: inferred from neighbouring sites
*R*[*F*^2^ > 2σ(*F*^2^)] = 0.045	H atoms treated by a mixture of independent and constrained refinement
*wR*(*F*^2^) = 0.144	*w* = 1/[σ^2^(*F*_o_^2^) + (0.0925*P*)^2^ + 0.059*P*] where *P* = (*F*_o_^2^ + 2*F*_c_^2^)/3
*S* = 1.10	(Δ/σ)_max_ < 0.001
3981 reflections	Δρ_max_ = 0.22 e Å^−^^3^
318 parameters	Δρ_min_ = −0.25 e Å^−^^3^
6 restraints	Extinction correction: *SHELXL97* (Sheldrick, 2008), Fc^*^=kFc[1+0.001xFc^2^λ^3^/sin(2θ)]^-1/4^
Primary atom site location: structure-invariant direct methods	Extinction coefficient: 0.007 (3)

### Special details

**Table:**

Geometry. All e.s.d.'s (except the e.s.d. in the dihedral angle between two l.s. planes) are estimated using the full covariance matrix. The cell e.s.d.'s are taken into account individually in the estimation of e.s.d.'s in distances, angles and torsion angles; correlations between e.s.d.'s in cell parameters are only used when they are defined by crystal symmetry. An approximate (isotropic) treatment of cell e.s.d.'s is used for estimating e.s.d.'s involving l.s. planes.
Refinement. Refinement of *F*^2^ against ALL reflections. The weighted *R*-factor *wR* and goodness of fit *S* are based on *F*^2^, conventional *R*-factors *R* are based on *F*, with *F* set to zero for negative *F*^2^. The threshold expression of *F*^2^ > σ(*F*^2^) is used only for calculating *R*-factors(gt) *etc*. and is not relevant to the choice of reflections for refinement. *R*-factors based on *F*^2^ are statistically about twice as large as those based on *F*, and *R*- factors based on ALL data will be even larger.

### Fractional atomic coordinates and isotropic or equivalent isotropic displacement parameters (Å^2^)

**Table:**

	*x*	*y*	*z*	*U*_iso_*/*U*_eq_	
N1	0.12979 (14)	0.51453 (15)	−0.18153 (10)	0.0466 (3)	
N2	−0.05854 (14)	0.30191 (14)	−0.26517 (10)	0.0482 (3)	
N3	−0.04327 (13)	0.62903 (14)	−0.06414 (9)	0.0407 (3)	
N4	−0.10826 (15)	0.91520 (15)	0.12949 (10)	0.0473 (3)	
N5	0.10055 (14)	0.91107 (14)	0.07405 (10)	0.0423 (3)	
C1	0.06996 (18)	0.29713 (18)	−0.30066 (12)	0.0491 (4)	
C2	0.0931 (2)	0.1845 (2)	−0.37465 (15)	0.0685 (5)	
H2B	0.0151	0.0957	−0.4100	0.082*	
C3	0.2354 (2)	0.2091 (2)	−0.39367 (18)	0.0796 (6)	
H3B	0.2528	0.1363	−0.4437	0.095*	
C4	0.3535 (2)	0.3401 (3)	−0.33982 (19)	0.0801 (7)	
H4B	0.4485	0.3517	−0.3538	0.096*	
C5	0.3335 (2)	0.4530 (2)	−0.26625 (15)	0.0652 (5)	
H5B	0.4126	0.5406	−0.2303	0.078*	
C6	0.18948 (18)	0.42972 (18)	−0.24846 (12)	0.0479 (4)	
C7	−0.01687 (16)	0.43359 (16)	−0.19367 (11)	0.0424 (4)	
C8	−0.11129 (17)	0.49146 (16)	−0.13058 (11)	0.0422 (4)	
C9	−0.25971 (18)	0.40738 (18)	−0.13928 (13)	0.0526 (4)	
H9A	−0.3028	0.3116	−0.1857	0.063*	
C10	−0.34091 (19)	0.46967 (19)	−0.07748 (14)	0.0575 (5)	
H10A	−0.4405	0.4162	−0.0816	0.069*	
C11	−0.27421 (18)	0.61135 (18)	−0.00955 (13)	0.0511 (4)	
H11A	−0.3277	0.6555	0.0326	0.061*	
C12	−0.12534 (16)	0.68670 (16)	−0.00542 (11)	0.0408 (4)	
C13	−0.04638 (16)	0.83768 (16)	0.06640 (11)	0.0409 (3)	
C14	0.00703 (18)	1.04751 (17)	0.18249 (11)	0.0464 (4)	
C15	0.0069 (2)	1.1702 (2)	0.25982 (14)	0.0621 (5)	
H15A	−0.0794	1.1716	0.2839	0.075*	
C16	0.1385 (2)	1.2887 (2)	0.29928 (15)	0.0689 (5)	
H16A	0.1412	1.3716	0.3511	0.083*	
C17	0.2687 (2)	1.2877 (2)	0.26338 (14)	0.0633 (5)	
H17A	0.3556	1.3705	0.2918	0.076*	
C18	0.27226 (19)	1.16824 (18)	0.18751 (13)	0.0536 (4)	
H18A	0.3589	1.1682	0.1636	0.064*	
C19	0.13926 (17)	1.04728 (17)	0.14830 (11)	0.0438 (4)	
O1	−0.44196 (15)	0.06635 (14)	−0.27630 (11)	0.0743 (4)	
O2	−0.28356 (15)	0.04486 (15)	−0.37563 (10)	0.0657 (4)	
C20	−0.7674 (3)	−0.3547 (3)	−0.4697 (2)	0.1110 (10)	
H20A	−0.8595	−0.3850	−0.4481	0.167*	
H20B	−0.7183	−0.4317	−0.4725	0.167*	
H20C	−0.7909	−0.3404	−0.5366	0.167*	
C21	−0.6624 (2)	−0.2079 (2)	−0.39388 (18)	0.0824 (7)	
H21A	−0.7139	−0.1315	−0.3902	0.099*	
H21B	−0.6411	−0.2228	−0.3263	0.099*	
C22	−0.5148 (2)	−0.1512 (2)	−0.42169 (14)	0.0611 (5)	
H22A	−0.4618	−0.2261	−0.4227	0.073*	
H22B	−0.5366	−0.1408	−0.4905	0.073*	
C23	−0.41213 (18)	−0.00337 (18)	−0.35032 (13)	0.0500 (4)	
O1W	−0.40511 (15)	0.92334 (16)	0.11934 (11)	0.0647 (4)	
O2W	0.32779 (16)	0.81969 (16)	−0.04472 (12)	0.0690 (4)	
H2	0.1745 (19)	0.6070 (13)	−0.1365 (11)	0.057 (5)*	
H1	0.156 (2)	0.873 (2)	0.0390 (14)	0.056 (5)*	
H2W1	−0.3168 (14)	0.920 (3)	0.1236 (19)	0.100 (8)*	
H2W2	0.350 (3)	0.885 (2)	−0.0747 (19)	0.116 (10)*	
H1W2	0.407 (2)	0.839 (3)	−0.0027 (17)	0.129 (12)*	
H1W1	−0.435 (3)	0.928 (3)	0.1717 (13)	0.106 (9)*	
H3	−0.228 (2)	0.1309 (16)	−0.3376 (16)	0.096 (8)*	

### Atomic displacement parameters (Å^2^)

**Table:**

	*U*^11^	*U*^22^	*U*^33^	*U*^12^	*U*^13^	*U*^23^
N1	0.0350 (7)	0.0439 (7)	0.0510 (7)	0.0049 (5)	0.0084 (6)	0.0045 (6)
N2	0.0399 (7)	0.0449 (7)	0.0514 (7)	0.0072 (6)	0.0116 (6)	0.0037 (6)
N3	0.0329 (6)	0.0430 (7)	0.0420 (6)	0.0082 (5)	0.0075 (5)	0.0094 (5)
N4	0.0398 (7)	0.0497 (7)	0.0466 (7)	0.0087 (6)	0.0125 (6)	0.0070 (6)
N5	0.0336 (7)	0.0437 (7)	0.0456 (7)	0.0076 (5)	0.0109 (5)	0.0089 (6)
C1	0.0409 (9)	0.0491 (9)	0.0513 (9)	0.0088 (7)	0.0121 (7)	0.0078 (7)
C2	0.0587 (11)	0.0579 (11)	0.0726 (12)	0.0080 (9)	0.0231 (9)	−0.0063 (9)
C3	0.0684 (13)	0.0722 (13)	0.0897 (14)	0.0193 (10)	0.0379 (11)	−0.0039 (11)
C4	0.0530 (12)	0.0789 (13)	0.1034 (16)	0.0164 (10)	0.0377 (11)	0.0069 (12)
C5	0.0414 (9)	0.0656 (11)	0.0781 (12)	0.0069 (8)	0.0204 (9)	0.0071 (10)
C6	0.0401 (8)	0.0490 (8)	0.0510 (9)	0.0116 (7)	0.0120 (7)	0.0094 (7)
C7	0.0351 (8)	0.0431 (8)	0.0445 (8)	0.0082 (6)	0.0065 (6)	0.0106 (6)
C8	0.0364 (8)	0.0405 (7)	0.0456 (8)	0.0082 (6)	0.0073 (6)	0.0104 (6)
C9	0.0399 (9)	0.0417 (8)	0.0638 (10)	0.0011 (7)	0.0123 (8)	0.0048 (7)
C10	0.0368 (8)	0.0498 (9)	0.0759 (12)	−0.0013 (7)	0.0209 (8)	0.0102 (8)
C11	0.0403 (9)	0.0485 (9)	0.0615 (10)	0.0072 (7)	0.0215 (7)	0.0104 (7)
C12	0.0337 (8)	0.0426 (8)	0.0441 (8)	0.0075 (6)	0.0092 (6)	0.0138 (6)
C13	0.0340 (8)	0.0431 (8)	0.0415 (8)	0.0064 (6)	0.0079 (6)	0.0114 (6)
C14	0.0438 (9)	0.0447 (8)	0.0432 (8)	0.0073 (7)	0.0080 (7)	0.0076 (7)
C15	0.0603 (11)	0.0585 (10)	0.0579 (10)	0.0134 (9)	0.0179 (8)	0.0011 (8)
C16	0.0769 (14)	0.0522 (10)	0.0569 (10)	0.0108 (9)	0.0056 (9)	−0.0058 (8)
C17	0.0569 (11)	0.0499 (9)	0.0618 (11)	−0.0010 (8)	−0.0030 (9)	0.0077 (8)
C18	0.0413 (9)	0.0513 (9)	0.0569 (10)	0.0023 (7)	0.0027 (7)	0.0138 (8)
C19	0.0387 (8)	0.0453 (8)	0.0416 (8)	0.0073 (6)	0.0041 (6)	0.0118 (7)
O1	0.0559 (8)	0.0539 (7)	0.0927 (10)	0.0011 (6)	0.0302 (7)	−0.0089 (7)
O2	0.0543 (8)	0.0667 (8)	0.0587 (7)	0.0007 (6)	0.0171 (6)	0.0021 (6)
C20	0.103 (2)	0.0735 (15)	0.1010 (18)	−0.0274 (14)	−0.0127 (15)	0.0078 (13)
C21	0.0645 (13)	0.0620 (12)	0.0910 (15)	−0.0090 (10)	0.0110 (11)	0.0033 (11)
C22	0.0641 (12)	0.0533 (9)	0.0529 (9)	0.0096 (9)	0.0011 (8)	0.0096 (8)
C23	0.0453 (9)	0.0477 (9)	0.0533 (9)	0.0119 (7)	0.0087 (7)	0.0123 (7)
O1W	0.0420 (7)	0.0799 (9)	0.0668 (8)	0.0146 (6)	0.0135 (6)	0.0150 (7)
O2W	0.0487 (8)	0.0607 (8)	0.0793 (9)	−0.0024 (6)	0.0019 (7)	0.0172 (7)

### Geometric parameters (Å, º)

**Table:**

N1—C7	1.358 (2)	C11—H11A	0.9300
N1—C6	1.375 (2)	C12—C13	1.470 (2)
N1—H2	0.90 (2)	C14—C15	1.393 (2)
N2—C7	1.3259 (19)	C14—C19	1.403 (2)
N2—C1	1.386 (2)	C15—C16	1.371 (3)
N3—C12	1.3350 (19)	C15—H15A	0.9300
N3—C8	1.3401 (19)	C16—C17	1.398 (3)
N4—C13	1.3203 (19)	C16—H16A	0.9300
N4—C14	1.383 (2)	C17—C18	1.373 (3)
N5—C13	1.3533 (19)	C17—H17A	0.9300
N5—C19	1.377 (2)	C18—C19	1.391 (2)
N5—H1	0.86 (2)	C18—H18A	0.9300
C1—C2	1.393 (2)	O1—C23	1.203 (2)
C1—C6	1.401 (2)	O2—C23	1.315 (2)
C2—C3	1.379 (3)	O2—H3	0.85 (2)
C2—H2B	0.9300	C20—C21	1.518 (3)
C3—C4	1.391 (3)	C20—H20A	0.9600
C3—H3B	0.9300	C20—H20B	0.9600
C4—C5	1.378 (3)	C20—H20C	0.9600
C4—H4B	0.9300	C21—C22	1.504 (3)
C5—C6	1.388 (2)	C21—H21A	0.9700
C5—H5B	0.9300	C21—H21B	0.9700
C7—C8	1.465 (2)	C22—C23	1.496 (2)
C8—C9	1.390 (2)	C22—H22A	0.9700
C9—C10	1.374 (2)	C22—H22B	0.9700
C9—H9A	0.9300	O1W—H2W1	0.83 (2)
C10—C11	1.374 (2)	O1W—H1W1	0.82 (2)
C10—H10A	0.9300	O2W—H2W2	0.82 (2)
C11—C12	1.385 (2)	O2W—H1W2	0.81 (2)
			
C7—N1—C6	107.54 (13)	N4—C13—N5	113.13 (13)
C7—N1—H2	123.1 (12)	N4—C13—C12	124.76 (13)
C6—N1—H2	129.3 (12)	N5—C13—C12	122.12 (13)
C7—N2—C1	104.86 (13)	N4—C14—C15	129.84 (16)
C12—N3—C8	117.15 (13)	N4—C14—C19	110.09 (13)
C13—N4—C14	104.64 (13)	C15—C14—C19	120.07 (16)
C13—N5—C19	107.30 (13)	C16—C15—C14	117.73 (18)
C13—N5—H1	123.4 (13)	C16—C15—H15A	121.1
C19—N5—H1	129.3 (13)	C14—C15—H15A	121.1
N2—C1—C2	130.11 (16)	C15—C16—C17	121.52 (17)
N2—C1—C6	109.96 (14)	C15—C16—H16A	119.2
C2—C1—C6	119.92 (16)	C17—C16—H16A	119.2
C3—C2—C1	117.73 (18)	C18—C17—C16	122.03 (17)
C3—C2—H2B	121.1	C18—C17—H17A	119.0
C1—C2—H2B	121.1	C16—C17—H17A	119.0
C2—C3—C4	121.61 (18)	C17—C18—C19	116.43 (17)
C2—C3—H3B	119.2	C17—C18—H18A	121.8
C4—C3—H3B	119.2	C19—C18—H18A	121.8
C5—C4—C3	121.71 (18)	N5—C19—C18	132.95 (15)
C5—C4—H4B	119.1	N5—C19—C14	104.84 (14)
C3—C4—H4B	119.1	C18—C19—C14	122.20 (15)
C4—C5—C6	116.72 (18)	C23—O2—H3	113.9 (17)
C4—C5—H5B	121.6	C21—C20—H20A	109.5
C6—C5—H5B	121.6	C21—C20—H20B	109.5
N1—C6—C5	132.63 (16)	H20A—C20—H20B	109.5
N1—C6—C1	105.07 (14)	C21—C20—H20C	109.5
C5—C6—C1	122.27 (16)	H20A—C20—H20C	109.5
N2—C7—N1	112.57 (13)	H20B—C20—H20C	109.5
N2—C7—C8	126.42 (14)	C22—C21—C20	113.3 (2)
N1—C7—C8	121.00 (13)	C22—C21—H21A	108.9
N3—C8—C9	123.26 (14)	C20—C21—H21A	108.9
N3—C8—C7	115.06 (13)	C22—C21—H21B	108.9
C9—C8—C7	121.67 (14)	C20—C21—H21B	108.9
C10—C9—C8	118.15 (15)	H21A—C21—H21B	107.7
C10—C9—H9A	120.9	C23—C22—C21	114.24 (16)
C8—C9—H9A	120.9	C23—C22—H22A	108.7
C9—C10—C11	119.67 (15)	C21—C22—H22A	108.7
C9—C10—H10A	120.2	C23—C22—H22B	108.7
C11—C10—H10A	120.2	C21—C22—H22B	108.7
C10—C11—C12	118.33 (15)	H22A—C22—H22B	107.6
C10—C11—H11A	120.8	O1—C23—O2	122.25 (16)
C12—C11—H11A	120.8	O1—C23—C22	124.19 (16)
N3—C12—C11	123.43 (14)	O2—C23—C22	113.56 (15)
N3—C12—C13	115.37 (13)	H2W1—O1W—H1W1	117 (3)
C11—C12—C13	121.19 (14)	H2W2—O2W—H1W2	102 (3)
			
C7—N2—C1—C2	178.8 (2)	C8—N3—C12—C13	179.95 (13)
C7—N2—C1—C6	−0.07 (19)	C10—C11—C12—N3	0.1 (3)
N2—C1—C2—C3	−178.9 (2)	C10—C11—C12—C13	−179.18 (15)
C6—C1—C2—C3	−0.1 (3)	C14—N4—C13—N5	−0.62 (17)
C1—C2—C3—C4	1.3 (4)	C14—N4—C13—C12	179.13 (14)
C2—C3—C4—C5	−1.3 (4)	C19—N5—C13—N4	0.37 (18)
C3—C4—C5—C6	0.0 (4)	C19—N5—C13—C12	−179.38 (13)
C7—N1—C6—C5	−177.18 (19)	N3—C12—C13—N4	178.66 (14)
C7—N1—C6—C1	0.76 (18)	C11—C12—C13—N4	−2.0 (2)
C4—C5—C6—N1	178.9 (2)	N3—C12—C13—N5	−1.6 (2)
C4—C5—C6—C1	1.2 (3)	C11—C12—C13—N5	177.75 (14)
N2—C1—C6—N1	−0.43 (19)	C13—N4—C14—C15	−178.98 (18)
C2—C1—C6—N1	−179.43 (17)	C13—N4—C14—C19	0.63 (17)
N2—C1—C6—C5	177.78 (16)	N4—C14—C15—C16	−179.71 (17)
C2—C1—C6—C5	−1.2 (3)	C19—C14—C15—C16	0.7 (3)
C1—N2—C7—N1	0.58 (18)	C14—C15—C16—C17	0.2 (3)
C1—N2—C7—C8	−178.33 (15)	C15—C16—C17—C18	−0.4 (3)
C6—N1—C7—N2	−0.87 (19)	C16—C17—C18—C19	−0.3 (3)
C6—N1—C7—C8	178.11 (14)	C13—N5—C19—C18	−179.13 (17)
C12—N3—C8—C9	−1.1 (2)	C13—N5—C19—C14	0.05 (17)
C12—N3—C8—C7	179.62 (13)	C17—C18—C19—N5	−179.70 (17)
N2—C7—C8—N3	−179.30 (14)	C17—C18—C19—C14	1.2 (2)
N1—C7—C8—N3	1.9 (2)	N4—C14—C19—N5	−0.42 (17)
N2—C7—C8—C9	1.4 (3)	C15—C14—C19—N5	179.24 (15)
N1—C7—C8—C9	−177.42 (15)	N4—C14—C19—C18	178.87 (14)
N3—C8—C9—C10	0.9 (3)	C15—C14—C19—C18	−1.5 (3)
C7—C8—C9—C10	−179.91 (15)	C20—C21—C22—C23	177.6 (2)
C8—C9—C10—C11	−0.1 (3)	C21—C22—C23—O1	0.2 (3)
C9—C10—C11—C12	−0.4 (3)	C21—C22—C23—O2	−179.94 (17)
C8—N3—C12—C11	0.6 (2)		

### Hydrogen-bond geometry (Å, º)

**Table:**

*D*—H···*A*	*D*—H	H···*A*	*D*···*A*	*D*—H···*A*
N1—H2···O2*W*	0.90 (2)	2.12 (1)	3.006 (2)	166 (2)
N5—H1···O2*W*	0.86 (2)	2.234 (19)	3.083 (2)	169.1 (17)
O1*W*—H2*W*1···N4	0.83 (2)	1.96 (1)	2.7901 (19)	178 (3)
O2*W*—H2*W*2···O1*W*^i^	0.82 (2)	2.04 (1)	2.852 (2)	168 (3)
O2*W*—H1*W*2···O1*W*^ii^	0.81 (2)	2.06 (1)	2.856 (2)	168 (3)
O1*W*—H1*W*1···O1^iii^	0.82 (2)	2.00 (1)	2.795 (2)	166 (3)
O2—H3···N2	0.85 (2)	1.89 (1)	2.712 (2)	164 (2)

Symmetry codes: (i) −*x*, −*y*+2, −*z*; (ii) *x*+1, *y*, *z*; (iii) −*x*−1, −*y*+1, −*z*.
